# Structural Characterization and Hypoglycemic Activity of a Novel Pumpkin Peel Polysaccharide-Chromium(III) Complex

**DOI:** 10.3390/foods11131821

**Published:** 2022-06-21

**Authors:** Wen Zhang, Lingyu Li, Yue Ma, Xiaole Chen, Tao Lan, Long Chen, Zhenjia Zheng

**Affiliations:** 1 Key Laboratory of Food Processing Technology and Quality Control of Shandong Higher Education Institutes, College of Food Science and Engineering, Shandong Agricultural University, 61 Daizong Street, Tai’an 271018, China; 18364705726@163.com (W.Z.); sdnydxlly@163.com (L.L.); c18854881522@163.com (X.C.); 2 Institute of Agri-Food Processing and Nutrition, Beijing Academy of Agriculture and Forestry Sciences, Beijing Key Laboratory of Agricultural Products of Fruits and Vegetables Preservation and Processing, Key Laboratory of Vegetable Postharvest Processing, Ministry of Agriculture and Rural Affairs, Beijing 100097, China; mayue@iapn.org.cn; 3 Sub-Institute of Agriculture and Food Standardization, China National Institute of Standardization, Beijing 100191, China; 4 Key Laboratory for Applied Technology of Sophisticated Analytical Instruments of Shandong Province, Shandong Analysis and Test Center, Qilu University of Technology (Shandong Academy of Sciences), Jinan 250014, China; chlong718@126.com

**Keywords:** pumpkin peel polysaccharide, chromium (III), structure, hypoglycemic activity

## Abstract

The aim of our study was to synthesize a pumpkin peel polysaccharide (PPP)-Cr(III) complex and investigate its hypoglycemic activity. Firstly, a novel PPP-Cr(III) complex with a Cr content of 23.77 mg/g was synthesized and characterized. Physicochemical characterization indicated that PPP-Cr(III) had some changes in chemical composition, monosaccharide composition, and morphological structure compared with PPP. The molecular weights of PPP-Cr(III) and PPP were 1.398 × 10^6^ g/mol and 3.386 × 10^6^ g/mol, respectively, showing a lower molecular weight after the introduction of Cr(III). Fourier transform infrared spectroscopy showed that a new characteristic absorption peak of Cr-O appeared at 534 cm^−1^ in PPP-Cr(III), indicating that Cr(III) was successfully complexed with PPP. Secondly, the hypoglycemic activity of PPP-Cr(III) based on α-glucosidase inhibitory and insulin resistance (IR)-HepG2 cells was evaluated. Compared with PPP, PPP-Cr(III) exhibited a more significantly α-glucosidase inhibitory activity. The IR-HepG2 cells confirmed an obvious increase in glucose consumption. Western blot analysis demonstrated that the treated IR-HepG2 cells were able to increase the protein levels of p-AMPK and p-GSK-3β, indicating that IR-HepG2 cells exerted hypoglycemic activity via the AMPK/GSK-3β signaling pathway. These results suggested that PPP-Cr(III) had good hypoglycemic activity, which could provide theoretical support for the development of novel hypoglycemic products.

## 1. Introduction

Pumpkin (*Cucurbita moschata*), belonging to the Cucurbitaceae family, is widely cultivated and consumed worldwide [1]. Pumpkin has been regarded as a nutritious and delicious vegetable or medicine, which can protect humans and animals from various diseases, and is found to show hypoglycemic, hypolipidemic, and antioxidant activities [2,3]. These functional properties are attributed to their rich components, such as polysaccharides, carotenoids, pectin, polyphenols, vitamins, and minerals [4,5]. Pumpkin fruit has an enormous comprehensive utilization value, and its pulp, seeds, and peel are all appropriate and valued edible resources. Pumpkin pulp is usually used for producing purees, jams, and sweets; seeds are used for roasted seeds, protein products, and edible oil, in which large amounts of peels are discarded as agricultural byproducts in food processing, resulting in resources waste and serious environmental pollution [6]. However, pumpkin peel might be an attractive material for developing functional foods due to its polysaccharides and carotenoids [7,8]. In recent years, polysaccharides obtained from pumpkins have received increasing attention. Bai et al., (2012) obtained a pumpkin polysaccharide named PP1, and it was extracted using hot water and purified by the cetyl trimethyl ammonium bromide method [9]. The structural analysis indicated that the molecular weight of PP1 was 1.51 × 10^5^ g/mol and mainly consisted of glucose, arabinose, galactose, and fructose with a molar ratio of 5.52:2.76:2.24:0.922, and the structure of PP1 might be a single chain with no or few branches by atomic force microscope. Wang et al., (2013) [10] reported the effects of different extraction methods (conventional, ultrasound-assisted, and microwave-assisted water extraction) on composition and molecular weight, and the highest extraction yield was found at 4.52% using microwave-assisted water extraction. The obtained polysaccharide structures were significantly affected by different extraction methods, in which three polysaccharides were mainly composed of glucose, arabinose, galactose, and fructose in different ratios, and the main molecular weights were 7.48 × 10^4^ g/mol, 8.83 × 10^4^ g/mol, and 1.042 × 10^5^ g/mol, respectively. Furthermore, the extraction, isolation, and biological activity of pectic polysaccharides from pumpkin peel have been reported previously [7]. So far, there are few reports about polysaccharides from pumpkin peel, and most of the studies focus on extraction and purification, while structural characteristics, biological evaluation, and mechanism of polysaccharides from pumpkin peel are still unclear. Therefore, it is very necessary to analyze the structural characteristics, biological evaluation, and mechanism of pumpkin peel polysaccharides by multiple techniques for industrial production conservatively and further scientific research.

Polysaccharides are important natural high-molecular-weight polymers and are non-toxic, inexpensive, and highly abundant. Previous studies have reported that polysaccharides have various bioactive activities such as hypoglycemic, antioxidant, and antitumor [4,5,7]. In recent years, polysaccharides have been widely used as a nanocarrier for oral delivery systems due to their low toxicity and high biocompatibility [11]. Furthermore, some studies have also confirmed that the biological activities of polysaccharides can be improved after chemical modification, such as selenium, chromium, zinc, and iron complex formation [12,13]. Chromium (Cr) is an essential component in humans and animals, which is involved in the metabolism of carbohydrates, proteins, lipids, and nucleic acids in the body [14]. In particular, Cr(III) can accelerate the utilization of glucose and improve insulin resistance [15]. Polysaccharide-Cr(III) complexes have shown the dual advantages of polysaccharides and Cr(III), and the decoration of polysaccharides can improve the bioactivity of Cr(III) and can better control diabetes [16]. Guo et al., (2020) [17] reported a novel *Grifola frondosa* polysaccharide-Cr(III), which can inhibit lipid accumulation and steatosis in the liver, modulate the intestinal microflora, and regulate the mRNA expression related to glucose and lipid metabolism. Zhang et al., (2019) [18] reported a *Momordica charantia* L. polysaccharide-Cr(III), which can decrease fasting blood glucose whereas improving the insulin levels and antioxidant enzyme activity in streptozotocin-induced diabetic mice. Therefore, it was hypothesized in the present study that the pumpkin peel polysaccharides after introducing chromium (III) would produce a synergistic effect of polysaccharides and Cr(III) to improve their hypoglycemic activities.

Hence, a novel pumpkin peel polysaccharide-chromium(III) complex was synthesized using a self-assembling reaction of PPP and chromium chloride (CrCl_3_). The chemical structure was analyzed by high-performance gel permeation chromatography (HPGPC), high-performance anion-exchange chromatography coupled with pulsed amperometric detector (HPAEC-PAD), ultraviolet-visible (UV-vis) spectra, Fourier transform infrared (FT-IR) spectra, circular dichroism (CD) spectra, X-ray diffraction (XRD), thermal analysis, scanning electron microscope (SEM), and transmission electron microscopy (TEM) analysis. Additionally, the hypoglycemic activity of PPP-Cr(III) was investigated by the insulin resistance (IR)-HepG2 cells model in vitro, and Western blot analysis was used to explore the hypoglycemic mechanism. The results provide the structural information and demonstrate their potential as novel hypoglycemic food supplements in the functional food and pharmaceutical industries, as well as improve industrial production conservatively.

## 2. Materials and Methods

### 2.1. Materials and Chemicals

Fresh pumpkin peel was obtained from Laiyang Mengyu Co., Ltd. (Yantai, China). CrCl_3_·6H_2_O (analytical grade) was obtained by Tianjin Kaitong chemical reagent Co., Ltd. (Shanghai, China). Trifluoroacetic acid (TFA) and insulin were provided by Sigma-Aldrich Co., Ltd. (St. Louis, MO, USA). α-Glucosidase, 4-nitrophenyl-α-D-glucopyranoside (PNPG), penicillin-streptomycin, and albumin bovine were purchased from Beijing Solarbio Technology Co., Ltd. (Beijing, China). Acarbose and monosaccharide standards (rhamnose, arabinose, galactose, glucose, mannose, xylose, fructose, galacturonic acid, and glucuronic acid) were obtained from Shanghai Yuanye Bio-Technology Co., Ltd. (Shanghai, China). Sodium acetate, anhydrous (electrochemical grade), and SS254-500 sodium hydroxide solution were purchased from Thermo Fisher Scientific (Sunnyrale, CA, USA). High-glucose DMEM medium was obtained from Wuhan Servicebio Technology Co., Ltd. (Wuhan, China). Fetal bovine serum was purchased from Zhejiang Tianhang Biotechnology Co., Ltd. (Huzhou, China). Trivalent chromium standard solution was supplied by Beijing Century Aoke Biotechnology Co. Ltd. (Beijing, China). All other chemicals and reagents used were of analytical grade.

### 2.2. Extraction and Purification of PPP

The extraction and purification of PPP were carried out according to the previous procedures with slight modifications [19]. Briefly, 1 kg of fresh pumpkin peel was extracted with 6 L distilled water at 100 °C for 90 min twice, and the supernatant was merged and concentrated to 2 L by a rotary evaporator under vacuum at 60 °C. Then, the proteins were removed several times with the Sevag reagent (chloroform/butyl alcohol = 4:1, *v*/*v*) until no more floccules were generated. Next, the crude pumpkin peel extract was precipitated by adding a four-fold volume of absolute ethanol and then kept at 4 °C overnight. The precipitate was obtained by centrifugation (4000 rpm, 10 min), followed by redissolution using distilled water and dialysis using a dialysis membrane with a molecular weight cut-off of 500 Da against distilled water for 72 h. Finally, the PPP was prepared by lyophilization with a freeze dryer.

### 2.3. Synthesis of PPP-Cr(III) Complex

PPP-Cr(III) complex was synthesized by a self-assembling reaction of PPP and CrCl_3_ with moderate modification [20]. Specifically, an equal volume of PPP solution (20 mg/mL) was mixed with CrCl_3_·6H_2_O (2.69 mg/mL), followed by adjusting the pH value from ~3.9 to 6.8 by 1 mol/L NaOH solution. Furthermore, the mixture was shaken in a water bath with a DKZ-2B shaking incubator (Shanghai Yiheng, Shanghai, China) at 150 rpm for 60 min at 60 °C. After cooling, a 4-fold volume of absolute ethanol was added to the mixture and then kept at 4 °C for 12 h. After centrifugation (4000 rpm, 10 min), the precipitate was redissolved in distilled water, followed by dialysis (molecular weight cut-off of 500 Da) and lyophilization. Finally, PPP-Cr(III) complex was obtained and stored in a 4 °C refrigerator for further study.

### 2.4. Physicochemical Properties of PPP-Cr(III) Complex

The total sugar content was determined using the phenol-sulfuric acid method at 490 nm with the glucose as standard [21], and the polysaccharide yield (%) was calculated using the following Equation (1). The protein content was determined by the Bradford method keeping the albumin bovine as standard [22].

(1) $$\mathrm{Polysaccharide}\;\mathrm{yield}\;\left(\%\right)=\frac{p\times m_{1}}{m_{2}}\times 100$$

where *p*(%) is the polysaccharide content calculated using the phenol-sulfuric acid method, *m*_1_(g) is the obtained polysaccharide weight after lyophilization, and *m*_2_(g) is the weight of fresh raw material.

### 2.5. The Content of Cr Element

The Cr content of PPP-Cr(III) was detected by inductively coupled plasma-mass spectrometry (ICP-MS) [23]. Briefly, 20 mg of sample was kept in 50 mL tubes, and then 7 mL of HNO_3_ was added and kept at 25 °C overnight. The ramp temperature was set at 120 °C for 5 min, and the temperature was maintained for 3 min, 180 °C for 5 min, and maintained the temperature for 10 min. After acid digestion and cooling, the sample was transferred to a 50-mL volumetric flask, and then 2% HNO_3_ was added to 50 mL, and the solution was mixed well. Then, the Cr content was detected by Agilent 7800 ICP-MS (Agilent, Santa Clara, CA, USA).

The radio frequency power was 1550 W, the Plasma and auxiliary gas flow rates were 15 and 0.99 L/min, respectively, and the helium flow rate was 5 mL/min. The spray chamber temperature was 2 °C. The Cr standard curve was built for quantitative analysis. The Cr(III) standard solutions were dissolved in 2% HNO_3_ (0.1, 0.2, 0.4, 0.6, 0.8, and 1.0 µg/mL), and then used for ICP-MS determination. Using the intensity as the vertical coordinate (Y) and the concentrations of the Cr standard solutions as the abscissa (X), the obtained linear calibration curve of Cr content and minimally admissible coefficient of determination (R^2^) was as Y = 32.1232X + 0.0063 (R^2^ = 0.9998).

### 2.6. Characterization of PPP-Cr(III) Complex

#### 2.6.1. Molecular Weight Analysis

The molecular weight (Mw) and polydispersity (Mw/Mn) distribution were analyzed using an HPGPC equipped with multiangle angle laser light scattering and refractive index detector (HPGPC-MALLS/RID) (Wyatt-Dawn Heleos-II, Santa Barbara, CA, USA) [24]. In short, the powdered sample (PPP-Cr(III) and PPP) was prepared as the 2 mg/mL solution and then filtered by a 0.22 μm millipore filter. Then, 200 μL of the sample solution was injected into a Shodex SB-806M HQ gel column (30 cm × 7.8 mm) at a column temperature of 40 °C. The mobile phase was 0.1 mol/L NaNO_3_ solution, and the flow rate was 0.5 mL/min. The detector temperature was 30 °C, and the refractive index increment was kept at 0.145 mL/g. The results were analyzed using the Astra 5.3 software (Wyatt Technology Corp., Santa Barbara, CA, USA).

#### 2.6.2. Monosaccharide Composition Analysis

The monosaccharide composition was analyzed according to the previous procedure in the laboratory using HPAEC-PAD (Thermo Fisher ICS-5000+, Waltham, MA, USA) [24]. Specifically, 10 mg of samples (PPP-Cr(III) and PPP) was hydrolyzed with 2 mL of 2.0 M TFA at 80 °C. After hydrolysis for 5 h, the remaining TFA of solutions was removed using a rotary evaporator at 60 °C until the pH value was 7.0. Then, the sample was dissolved in 10 mL of ultra-pure water, and clarification using Supelclean™ ENVI-18 SPE tube (500 mg/6mL) (Merck, St. Louis, MO, USA) was carried out. Finally, 100 μL of clarification sample was dissolved in 4 mL ultra-pure water, followed by purification using a 0.22 μm millipore filter. The Dionex™ AminoPac™ PA10 IC column (2 × 250 mm) was used to analyze the monosaccharide profile with a column temperature of 30 °C. The mobile phase was 0.20 mol/L NaOH and 0.1 mol/L NaAc solution with a flow rate of 0.250 mL/min. Monosaccharide composition and molar ratio were analyzed based on retention time and peak area with a series of monosaccharide standards.

#### 2.6.3. UV-Vis and FT-IR Spectra Analysis

The UV spectra of PPP and PPP-Cr(III) (1 mg/mL) were measured by a UV-Vis spectrophotometer (UV-2450, Shimadzu, Tokyo, Japan), and the wavelength range was 190–800 nm. Additionally, the FT-IR spectra were recorded using a Nicolet 6700 FT-IR spectrophotometer (Thermo Fisher Scientific, Waltham, MA, USA). Specifically, the potassium bromide (KBr) was dried at 105 °C for 5 h, followed by a 2 mg freeze-dried sample mixed with 200 mg KBr were fully ground into power in the agate grinder, and then the tablet was pressed in the tablet press, the pressure was 2 tons, and time was 2 min [25]. This analysis was operated from the range from 4000^−1^ to 400 cm^−1^ at a resolution of 4 cm^−1^ in triplicate. The OMNIC 8.2 software was used for data collection and annotation.

#### 2.6.4. CD Spectra Analysis

The CD spectra of PPP and PPP-Cr(III) were acquired on a Chirascan-V100 circular dichroism spectrometer (Applied Photophysics Ltd., Surrey, UK) [26]. The sample was prepared as 1 mg/mL and measured over the wavelength range of 180–450 nm at 25 °C using a quartz cuvette with a path length of 0.1 cm. The scan rate, bandwidth, and time constant were set as 100 nm/min, 1.0 nm, and 0.5 s, respectively.

#### 2.6.5. XRD Analysis

The XRD pattern of PPP and PPP-Cr(III) were analyzed at 25 °C on an EMPYREAN X-ray diffractometer (PANalytical, Almelo, The Netherlands). The data were collected in the 2θ ranges of 5–80° with a step size of 0.01° and a counting times of 5 s/step.

#### 2.6.6. Thermal Analysis

Thermal gravity (TG), derivative thermal gravity (DTG), and differential scanning calorimetry (DSC) were conducted using a STA6000 simultaneous thermal analyzer (PerkinElmer, Waltham, MA, USA). Briefly, 10 mg of PPP and PPP-Cr(III) powder was heated from 30 °C to 600 °C, respectively. The data were collected at 10 °C/min of heating rates under a nitrogen (99.9%) atmosphere (50 mL/min).

### 2.7. Hypoglycemic Activity

#### 2.7.1. α-Glucosidase Inhibitory Activity

The α-glucosidase inhibitory effect was evaluated according to the reported method with some modifications [27]. The sample solution (0.2, 0.6, and 1.0 mg/mL) was prepared by phosphate buffer (1 mol/L, pH 6.9). Then, 100 μL of sample solutions were mixed with 100 μL α-glucosidase solution (0.25 U/mL) in a 96-well microplate. After shaking the mixture at 37 °C for 10 min, and then adding 100 μL of 4 mol/L PNPG (dissolved in phosphate buffer, pH 6.9), and incubated at 37 °C for 30 min. Finally, the reaction was stopped by adding 1 mL Na_2_CO_3_ (1 mol/L), and the solution absorbance was measured at 405 nm. Acarbose served as the positive control. The inhibition activity was obtained using Equation (2):

(2) $$\mathrm{Inhibition}\;\mathrm{activity}\;\left(\%\right)=\left(1-\frac{A_{j}-A_{k}}{A_{i}}\right)\times 100$$

where *A_i_* is the absorbance of the mixture of α-glucosidase and PNPG solution; *A_j_* is the absorbance of the mixture of the sample solution, α-glucosidase, and PNPG; *A_k_* is the absorbance of the mixture of PNPG and the sample solution.

#### 2.7.2. Hypoglycemic Activity in HepG2 Cells Model

##### 2.7.2.1. Cell Culture of HepG2 Cells

HepG2 cells cryopreserved in the refrigerator at −80 °C were removed and thawed at 37 °C in a water bath. Then, the HepG2 cells were transferred into a sterile centrifugal tube, and a 4 mL serum-free medium was added. After centrifugation at 1000 rpm for 5 min, the supernatant medium was removed, and 6 mL of complete culture medium (89% high-glucose DMEM medium, 10% fetal bovine serum, and 1% penicillin-streptomycin) was added to the tube and mixed fully. Afterward, the medium containing cells was transferred to a T25 culture bottle (Thermo Fisher, Suzhou, China) and cultured in an HF90 incubator (Heal Force, Shanghai, China) at 37 °C containing 5% CO_2_. After 80% confluence growth, the supernatant medium was removed, and 3 mL of PBS was added for washing and then removed. Then, HepG2 cells were harvested using 2 mL of 0.25% trypsin and sub-cultured onto a culture bottle in a complete medium at 37 °C containing 5% CO_2_.

##### 2.7.2.2. HepG2 Cells Viability Assay

After being sub-cultured twice, the cells were diluted to 1.0×10^5^ cell/mL (counted using an Eclipse Ts 2 inverted microscope, Nikon, Japan), and 100 μL of HepG2 cells was seeded in a 96 well culture plate and cultured in an incubator at 37 °C containing 5% CO_2_ and incubated for 24 h. Then, 100 μL of PPP or PPP-Cr(III) solutions (25, 50, 100, and 200 μg/mL) was added to each well and incubated for 24 h. Afterward, 50 μL of MTT solution (1 mg/mL) was added to each well and incubated in the dark for 4 h. Finally, DMSO was used to dissolve the formazan after removing MTT solutions, and the solution absorbance was measured at 450 nm. The cell viability was calculated as follows in Equation (3):

(3) $$\mathrm{Cell}\;\mathrm{viability}\;\left(\%\right)=\frac{A_{sample}-A_{blank}}{A_{control}-A_{blank}}\times 100$$

where *A_sample_*, *A_blank_*, and *A_control_* were absorbance of the group with samples, the blank group, and the group without samples, respectively.

##### 2.7.2.3. Evaluation of the Glucose Consumption Capacity in Insulin Resistance (IR)-Hep G2 Cells

Briefly, 100 μL of HepG2 cells in an exponential growth period were seeded into 96-well culture plates at a density of 1.0 × 10^5^ cell/mL and incubated in a complete medium for 24 h. Subsequently, the supernatant medium was discarded, followed by 100 μL of insulin (10^−6^ mol/L) was added and incubated for 36 h to establish the IR-HepG2 cells model (except for the control group). Then, the cells of the control group (HepG2) and model group (IR-HepG2) were cultured in serum-free DMEM for 12 h for starvation treatment, followed by washing twice with PBS buffer after removing the medium. Then, 200 μL of PPP and PPP-Cr(III) solutions (25, 50, 100, and 200 μg/mL) was added to each well for 24 h, and glucose contents were measured by a glucose assay kit (Nanjing Jiancheng Bioengineering Institute, Nanjing, China) following the manufacturer’s approach, and the absorbance was measured at 450 nm. The results were expressed as % of the glucose consumption capacity.

##### 2.7.2.4. Western Blot Analysis

The Western blot was done using the reported method with moderate modification [28,29]. HepG2 and IR-HepG2 cells were cultured as described in Section 2.7.2.1 and Section 2.7.2.3. Then, the total proteins were prepared from the cellular lysates, and the protein contents were determined using a Bio-Rad protein assay kit to ensure equal amounts of proteins. Afterward, the protein was separated using 10% SDS-polyacrylamide gel electrophoresis (prepared using an SDS-polyacrylamide gel kit) and transferred onto 0.45 μm polyvinylidene fluoride (PVDF) membranes at 250 mA for 2 h. The 0.45 μm PVDF membranes were blocked with 5% skim milk powder in TBST buffer at room temperature for 1–2 h, followed by incubation with primary antibodies (p-AMPK, p-GSK-3β, and β-Tublin) at 4 °C for 12 h, and then incubated with horseradish peroxidase-conjugated secondary antibodies (Goat Anti Rabbit IgG-HRP, Wuhan Servicebio Technology, Wuhan, China) at room temperature for 1–2 h. After each antibody incubation, the members were washed twice with TBST for 10 min each time. Finally, the prepared membranes were exposed to the ECL plus kit (ECL1/ECL2 = 1/1) for 1 min. The protein bands were visualized using a ChemiScope 6000 Touch fluorescence/chemiluminescence imaging system (Clinx Science Instruments, Shanghai, China) and measured (using an Image J 1.53k software, National Institutes of Health, Bethesda, MD, USA) to obtain the optical density ratio of the target proteins.

### 2.8. Statistical Analysis

All experiments were conducted in triplicate. All analysis data were presented as mean ± standard deviation. The significant differences were evaluated using a one-way analysis of variance (ANOVA) followed by the Waller-Duncan (W) test using SPSS (Version 21.0 Armonk, NY, USA), where *p* < 0.05 was assumed to be statistically significant.

## 3. Results and Discussion

### 3.1. Physicochemical Properties

In this work, the polysaccharide yield of PPP was 3.10% in fresh pumpkin peel. The physicochemical properties of PPP and PPP-Cr(III) are shown in Table 1. The total saccharides contents of PPP and PPP-Cr(III) were 77.79% and 61.16%, respectively, indicating that saccharides were the major components in PPP and PPP-Cr(III). Additionally, the total saccharides of samples were decreased due to the massive introduction of Cr(III). Moreover, The Cr content of PPP-Cr(III) was determined as 23.77 mg/g, indicating that PPP has a good ability to chelate Cr(III). These results indicated that the introduction of CrCl_3_ changed the total saccharides and Cr contents, which confirmed that PPP-Cr(III) was synthesized successfully. Furthermore, the protein contents of PPP and PPP-Cr(III) were 1.18% and 1.13%, indicating most of the proteins were removed completely by the Sevag reagent. Moreover, the surface and inner morphological structure were evidently different between PPP and PPP-Cr(III) by SEM and TEM results (Text S1 and Figure S1) [30,31].

### 3.2. Monosaccharide Composition

Monosaccharide composition is an important factor influencing the physicochemical and bioactivities of heteropolysaccharides. Both PPP and PPP-Cr(III) were composed of the same monosaccharide types but in different ratios. As shown in Table 1, PPP was composed of rhamnose, arabinose, galactose, glucose, and galacturonic acid with the molar ratio percentages of 0.52%, 2.24%, 4.81%, 86.24%, and 6.20%, respectively. Likewise, PPP-Cr(III) was composed of monosaccharides with the molar ratio percentages of 0.27%, 1.72%, 3.53%, 89.63%, and 4.86%. After modification, a slight increasing ratio in glucose was observed, while some other monosaccharide ratios (rhamnose, arabinose, galactose, and galacturonic acid) were decreased slightly. Therefore, the above results showed that the introduction of Cr(III) for PPP could cause some changes in the monosaccharide composition of pumpkin peel polysaccharides.

### 3.3. Molecular Weight

Molecular weight is considered as an important index for structural analysis of polysaccharides and polysaccharide complex because of its critical effect on structure-property relations. Interestingly, the molecular weight (Mw) of PPP-Cr(III) and PPP were 1.398 × 10^6^ g/mol and 3.386 × 10^6^ g/mol, respectively, which showed lower Mw after the introduction of Cr(III). Similar results were reported in the previous study; the Mw (7.348 × 10^4^ g/mol) of the polysaccharides extracted from *Artemisia sphaerocephala* (ASP) was larger than its selenized derivative (4.363, 1.141, 1.026, 2.268, 1.426, and 5.407 × 10^4^ g/mol), which might be due to the increasing HNO_3_ concentration and higher temperature accelerate the hydrolysis rate [32]. Likewise, it was found that pumpkin polysaccharides obtained an alkaline environment displayed the lower Mw due to *β*-elimination reactions by specifically cleaving the glycosidic linkages adjacent to methoxy groups of galacturonic acid (GalA) units [33]. However, Zhang et al., (2020) reported that the relative molecular weight of *Fritillaria ussuriensis* polysaccharide remains unchanged basically after zinc modification [34]. Jia et al., (2021) reported that the Mw of corn silk polysaccharide was significantly increased compared with its iron, zinc, or chromium complexes, which showed that metal ions, like a binder, promoted the connection of polysaccharide [13]. In this work, the initial pH was ~3.9, and the final pH was 6.8 using a 1 mol/L NaOH solution, which may be due to introducing NaOH solutions to cleave the glycosidic linkages adjacent to methoxy groups of GalA units of polysaccharides to decrease Mw. Additionally, this difference in molecular weight may be related to the characteristics of different polysaccharides and their complexes [13].

### 3.4. UV-Vis and FT-IR Spectra Analysis

**Figure 1:**
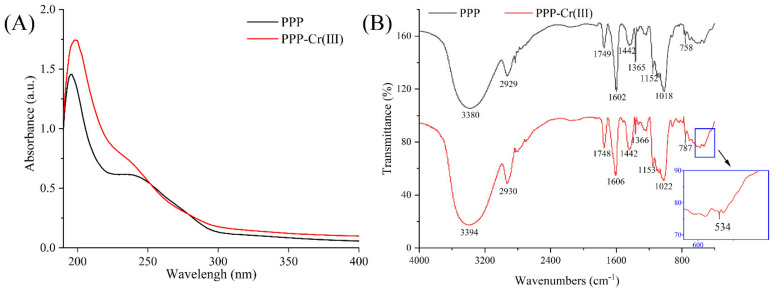
UV-vis (**A**) and FT-IR (**B**) spectra of PPP and PPP-Cr(III).

The UV-vis spectra of PPP and PPP-Cr(III) are shown in Figure 1A. There were no significant absorption peaks at 260–280 nm, indicating that few nucleic acids and proteins existed in PPP and PPP-Cr(III), and this result was consistent with the determination of protein contents (Table 1). In addition, the absorbance of PPP-Cr(III) solution was higher than PPP solution in the range of 200–250 nm, indicating that O-H and C=O in polysaccharides maybe participated in the complex formation with Cr(III) and oxygens might be the ligating atoms between Cr(III) and polysaccharides (o→Cr and oxo→Cr) (Figure 1A) [35,36]. In contrast, the absorbance of Cr(III) and corn silk polysaccharide complex was decreased distinctly, which may be because the complex of corn silk polysaccharide and Cr(III) caused a reduction in the conjugated system [13]. Thus, it is speculated that the complexing forms of Cr(III) and different polysaccharides are different.

The FT-IR spectra of PPP and PPP-Cr(III) are shown in Figure 1B. The strong peaks at 3380 cm^−1^ and 2929 cm^−1^ are attributed to O-H bond stretching vibration and C-H bond stretching vibration of the -CH_2_ groups, respectively [18]. The peaks at 1749 cm^−1^ were attributed to C=O stretching vibration, indicating the presence of uronic acid in PPP. The absorptions at 1606 cm^−1^ and 1442 cm^−1^ were anti-symmetrical C=O stretching vibration and symmetrical C=O stretching vibration of ionic carboxyl groups (i.e., −COOH^−^) [37]. Overall, the FT-IR spectra of PPP exhibited a typical polysaccharide structure. After the introduction of Cr(III), most of the characteristic peaks were similar to PPP, demonstrating that the main functional groups were not changed after complexation with Cr(III). However, the peaks of stretching vibration of O-H showed a red shift from 3380 to 3394 cm^−1^, indicating the formation of a coordination bond between O-H and Cr(III) [13]. Furthermore, the new characteristic absorption peak of Cr-O appeared at 534 cm^−1^ in PPP-Cr(III), showing that Cr(III) participated in the complexation reaction [18]. These results suggested that a novel PPP-Cr(III) was synthesized successfully, and they are consistent with UV-vis spectra results.

### 3.5. CD Spectra

**Figure 2:**
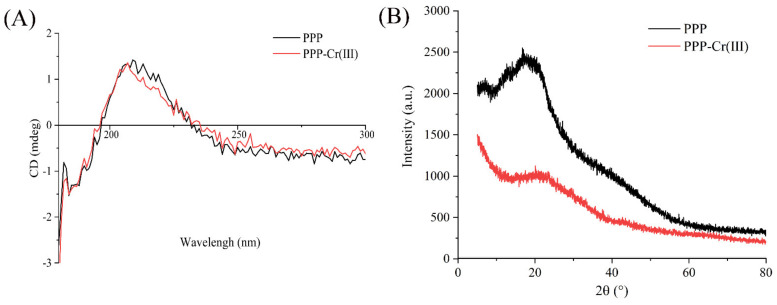
CD spectra (**A**), XRD pattern (**B**) of PPP and PPP-Cr(III).

The spatial conformational changes in the structure of macromolecules could be investigated by CD spectra [38]. It was reported that 210 nm and 190 nm have assigned to n→π* and π→π* in CD spectra of glycosaminoglycans, and carboxyl exhibited considerable optical activity [39]. As shown in Figure 2A, the main positive and negative Cotton effects of PPP are around 209 and 184 nm, indicating that there existed carboxyl groups in PPP. After the introduction of Cr(III) in PPP, corresponding signals changed slightly in CD spectra, which showed that basic polysaccharides chains were not changed after the introduction of Cr(III). Furthermore, the positive Cotton effect of PPP-Cr(III) shifted from 209 nm to 207 nm, indicating that the spatial configuration has changed between Cr(III) and the carboxyl group of polysaccharides [39].

### 3.6. XRD Pattern Analysis

The crystal structure characteristics between PPP and PPP-Cr(III) were compared by XRD pattern. As shown in Figure 2B, both PPP and PPP-Cr(III) had a broad diffraction peak at 2θ ≈ 20°, suggesting that PPP was typical amorphous material and the complex of PPP with Cr(III) did not change its crystallinity [13]. The diffraction pattern of PPP-Cr(III) had broad diffraction at 2θ ≈ 21° and PPP at 2θ ≈ 18°, indicating that PPP-Cr(III) forms a new amorphous structure. Additionally, this amorphous structure is attributed to hydrogen bonding interactions between O-H groups of polysaccharide chains [17], which is also consistent with the results of O-H groups of polysaccharides by UV-vis and FT-IR spectra.

### 3.7. Thermal Analysis

**Figure 3:**
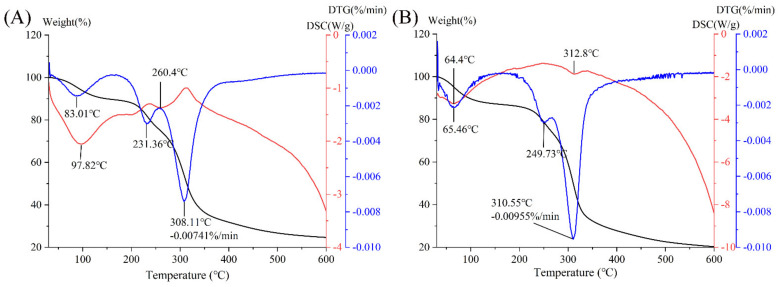
Thermal analysis of PPP (**A**) and PPP-Cr(III) (**B**).

The thermal stabilities of PPP and PPP-Cr(III) were studied by TG, DTG, and DSC. As shown in Figure 3A,B, the TG curve of PPP had two stages, namely 10.32% (30–160 °C) caused by loss of free water and partial bound water, and 65.06% (160–600 °C) caused by loss of polysaccharide. By comparison, the TG curve of PPP-Cr(III) was divided into two stages, including 14.01% (30–200 °C) and 65.71% (200–600 °C). As shown in the DTG curve, the maximum weight loss rate was 0.00741%/min for PPP at 308.11 °C, while the PPP-Cr(III) was 0.00955%/min at 310.55 °C. In addition, the curve was stabilized after 600 °C, and the residual weight was 24.61%, while PPP-Cr(III) was 20.28%. Additionally, the DSC curve of PPP showed that there were two endothermic peaks at 97.82 °C and 260.4 °C, while the PPP-Cr(III) at 64.4 °C and 312.8 °C. From these results, both PPP and PPP-Cr(III) have good thermal stability, while the thermal stability was slightly decreased after complexing Cr(III), which was consistent with the results of Zhao et al. [40] and Gao et al. [12]. In the polysaccharide-Cr(III) complex, the O-H groups in the natural polysaccharide could combine with CrCl_3_ to form Cr-O groups [41]. However, the high temperature may cause the departure of Cr-O groups and then lead to the faster degradation of the polysaccharide-Cr(III) complex.

### 3.8. Hypoglycemic Activity

#### 3.8.1. α-Glucosidase Inhibitory Activity

**Figure 4:**
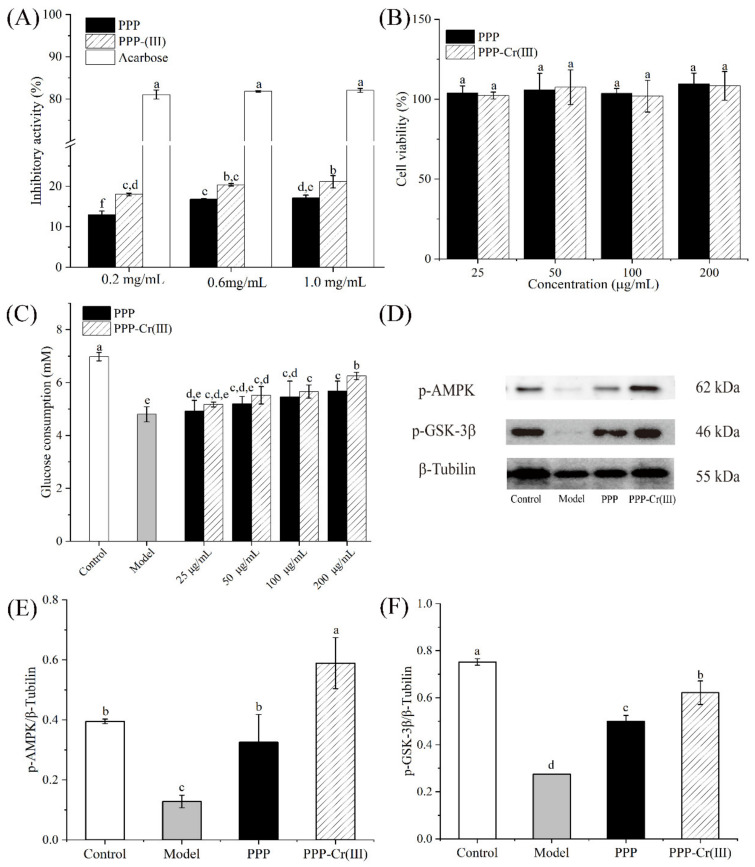
Hypoglycemic activities of PPP and PPP-Cr(III). Inhibition activity on α-glucosidase (**A**); effect on the cell viability (**B**); glucose consumption (**C**); Western blot bands of p-AMPK and p-GSK-3β (**D**); protein expression level of p-AMPK/β-Tubilin (**E**), and p-GSK-3β/β-Tubilin (**F**). The different letters above the adjacent bars indicate significant differences (*p* < 0.05, *n* = 3), while the same letter indicates no significant differences.

α-Glucosidase is a key enzyme in adjusting the blood glucose level, and α-glucosidase activity inhibitors are effective components in treating type II diabetes [42]. As shown in Figure 4A, the inhibitory effect of PPP and PPP-Cr(III) on α-glucosidase were dose-dependently increased in the range of 0.2–1.0 mg/mL; however, the positive control (acarbose) showed a significantly better inhibitory activity than PPP and PPP-Cr(III). The best α-glucosidase inhibitory activities of PPP and PPP-Cr(III) reached 17.09% and 21.11% at a concentration of 1.0 mg/mL, respectively. In the present study, the α-glucosidase inhibitory activity of PPP-Cr(III) was significantly higher than PPP from 0.2 to 1.0 mg/mL (*p* < 0.05), and PPP-Cr(III) exhibited a better α-glucosidase inhibitory.

#### 3.8.2. Cell Viability

HepG2 cells were incubated with PPP and PPP-Cr(III) (25–200 μg/mL) for 24 h, and the MTT agent was used to determine the toxicity by cell viability assay. As a result, PPP and PPP-Cr(III) did not exhibit significant cytotoxicity influence on HepG2 cells at different concentrations (25, 50, 100, and 200 μg/mL), indicating that PPP and PPP-Cr(III) could be used for the following bioactivity evaluation (Figure 4B).

#### 3.8.3. Glucose Consumption in IR-HepG2 Cells

Insulin resistance (IR) is another major cause of type II diabetes. It was mainly manifested as a decrease in insulin sensitivity of humans and animals, leading to the decline of glucose uptake and utilization, so excessive insulin was required to maintain healthy physiological functions. The HepG2 cells are widely applicable to screen drugs and functional components for IR treatment and mechanism study of hypoglycemic activity in vitro. It has been reported that polysaccharides could affect glucose uptake and increase the glucose consumption of IR-HepG2 cells [43]. In this work, an IR-HepG2 cells model was established to evaluate whether PPP and PPP-Cr(III) exert hypoglycemic activity through accelerating glucose uptake and utilization. As shown in Figure 4C, the glucose consumption of the model group (4.8 mM) treated with insulin decreased significantly compared with the control group (6.98 mM), indicating that the IR-HepG2 cells model was established successfully. Compared with the model group, the glucose consumption in IR-HepG2 cells was improved in a dose-dependent manner after treatment with PPP and PPP-Cr(III). At a concentration of 200 μg/mL, glucose consumption treated with PPP-Cr(III) (6.25 mM) improved significantly compared with PPP (5.68 mM), which was nearly 1.3-fold and 1.2-fold higher than the model group, suggesting that both two samples can promote glucose expenditure. Furthermore, the results showed that PPP-Cr(III) had a better capacity to improve glucose consumption in IR-HepG2 cells than PPP within the tested concentrations. Collectively, these results showed that PPP and PPP-Cr(III) could accelerate glucose metabolism in IR-HepG2 cells to exert hypoglycemic activity.

#### 3.8.4. PPP and PPP-Cr(III) Stimulated p-AMPK/p-GSK-3β Express in IR-HepG2 Cell Culture

To investigate the hypoglycemic mechanism of PPP and PPP-Cr(III), the phosphorylation levels of AMPK and GSK-3β were evaluated. AMPK was a heterotrimer composed of one catalytic subunit (α) and two regulatory subunits (β and γ), which can regulate insulin sensitivity and improve the glucose uptake of cells and the translocation of the glucose transporter (GLUT) [28,44]. Both PPP and PPP-Cr(III) could promote AMPK phosphorylation (p-AMPK), as seen in Figure 4D,E. Compared with the control group, the model group was significantly lower p-AMPK levels by IR-HepG2 cells (*p* < 0.05). Furthermore, both PPP and PPP-Cr(III) could significantly reverse the p-AMPK levels compared with control cells at 100 μg/mL (Figure 4D,E). This result suggested that AMPK was supposed to be a key protein in the carbohydrate metabolism signal transduction pathway targeted by PPP and PPP-Cr(III), especially PPP-Cr(III) exhibited better upregulation p-AMPK expression, and its level was significantly higher than the control group (*p* < 0.05).

Likewise, GSK-3β is a key protein for the IRS-1/Akt signaling pathway, which can reduce glycogen synthesis, and GSK-3β phosphorylation (p-GSK-3β) also plays a key role in insulin resistance [28]. Ye et al., (2019) [45] reported that sulfated rhamnose polysaccharide-Cr(III) complex could regulate hepatic glycogen synthesis and improve glucose metabolism by regulating the insulin-mediated PI3K/PKB/GSK-3β and PI3K/PKB/GLUT4 signaling pathway. In the present study, the IR-HepG2 cells model group downregulated p-GSK-3β expression (*p* < 0.05) compared with the control group (Figure 4D,F). Additionally, both PPP and PPP-Cr(III) significantly reversed the p-GSK-3β expression levels compared with control group cells at 100 μg/mL, and PPP-Cr(III) exhibited better upregulation of p-GSK-3β expression than PPP (*p* < 0.05) (Figure 4D,F).

It showed that PPP-Cr(III) improved the glucose uptake of IR-HepG2 cells by inducing the phosphorylation of AMPK and the up-regulation of the expressions of p-GSK-3β to improve insulin resistance [44,46]. These results explained why PPP and PPP–Cr(III) could accelerate glucose absorption and metabolism in the absence of insulin or with relatively low insulin production.

### 3.9. Structure-Hypoglycemic Activity of PPP-Cr(III)

According to the results of hypoglycemic activity in the α-glucosidase inhibitory and IR-HepG2 cells model described above, PPP and PPP-Cr(III) showed different hypoglycemic activities. Especially, PPP-Cr(III) exhibited better hypoglycemic activities than PPP in the α-glucosidase inhibitory and IR-HepG2 cells model. This high hypoglycemic activity might be related to their structural characteristics, including molecular weight, monosaccharide composition, glycosidic linkage, conformation, branching degrees, and functional groups [47,48]. Additionally, Deng et al., (2020) [49] reported that high content of galactose, arabinose, rhamnose, and galacturonic acid can enhance the α-glucosidase inhibitory. As for monosaccharide composition, arabinose, galactose, rhamnose, and galacturonic acid from PPP and PPP-Cr(III) may play a certain role in α-glucosidase inhibitory activity; however, interestingly, arabinose, galactose, galacturonic acid, and rhamnose were decreased slightly, and glucose was increased after the introduction of Cr(III) [49]. Xu et al., (2018) [50] reported that the polysaccharide from blackcurrant fruits with lower molecular weight exhibited higher α-glucosidase inhibitory. In the current study, the better α-glucosidase inhibitory of PPP-Cr(III) might be attributed to a relatively low molecular weight (1.398 × 10^6^ g/mol) than PPP (3.386 × 10^6^ g/mol), whereas further studies of the α-glucosidase inhibitory mechanism of PPP-Cr(III) should be performed.

In this study, glucose consumption of IR-HepG2 cells was significantly improved after treatment with PPP and PPP-Cr(III), and PPP-Cr(III) showed better ability. Additionally, the hypoglycemic mechanism of PPP and PPP-Cr(III) in IR-HepG2 cells regulated p-AMPK and p-GSK-3β expression to improve insulin resistance, and PPP-Cr(III) exhibited better upregulation. Comparing the structure of two polysaccharides, the basic polysaccharide chains of PPP were not changed after introduced of Cr(III) while forming new Cr-O bonds (o→Cr and oxo→Cr) in PPP-Cr(III). In the previous study, the sulfated rhamnose polysaccharides after the introduction of Cr(III) enhanced glucose metabolism by regulating the insulin-mediated PI3K/PKB/GSK-3β [45]. Cr(III) also could enhance insulin responsiveness by the AMPK signaling pathway [51]. These results suggested that Cr(III) in PPP-Cr(III) had a strong hypoglycemic activity.

Molecular weight is also an important factor affecting hypoglycemic activity. Deng et al., (2020) [52] obtained four konjac glucomannan with different molecular weights, which reported that medium molecular weights (7.57 × 10^5^ g/mol and 2.52 × 10^5^ g/mol) showed better effects on hypoglycemic effects than high or low molecular weight (1.1295 × 10^6^ g/mol and 8.73 × 10^4^ g/mol). Molecular weight could affect solubility, digestibility, metabolism, and bioavailability, and low molecular weight polysaccharide could be absorbed by cells and intestines into the bloodstream [2]. In this work, the lower molecular weight in PPP-Cr(III) had better hypoglycemic activity, which might be that low-molecular-weight PPP-Cr(III) was easier to enter cells or high solubility was better for repairing the surface damage of IR-HepG2 cells.

## 4. Conclusions

In the present study, a novel pumpkin peel polysaccharide-chromium(III) complex (PPP-Cr(III)) was synthesized successfully through the chelation of pumpkin peel polysaccharide and chromium(III). Various structural characterizations showed that Cr content was 23.77 mg/g, and important binding sites between PPP and PPP-Cr(III) were O-H and C=O. PPP-Cr(III) had some changes in chemical composition, monosaccharide composition, molecular weight, and morphological structure after the introduction of Cr(III). Compared with PPP, PPP-Cr(III) exhibited better hypoglycemic activity on α-glucosidase inhibitory and insulin resistance (IR)-HepG2 cells. Western blot analysis demonstrated that the treated IR-HepG2 cells were able to increase p-AMPK and p-GSK-3β expression, and PPP-Cr(III) might be mediated by the regulation of the AMPK/GSK-3β signaling pathway. Certainly, the in-depth mechanism of hypoglycemic activity of PPP-Cr(III) needs further exploration through the animal model and its effects on intestinal microflora. In summary, these results suggested that Cr(III) modification could improve the hypoglycemic bioactivity of PPP and could be used for hypoglycemic supplements in functional food.

## Figures and Tables

**Table 1:** Chemical composition and monosaccharide composition of PPP and PPP-Cr(III).

Samples	PPP	PPP-Cr(III)
Total saccharides content (%)	77.79 ± 4.18	61.16 ± 1.17
Protein content (%)	1.18 ± 0.05	1.13 ± 0.05
Chromium content (mg/g)	-	23.77 ± 0.39
Monosaccharides composition (molar ratio, %)		
Rhamnose	0.52	0.27
Arabinose	2.24	1.72
Galactose	4.81	3.53
Glucose	86.24	89.63
Galacturonic acid	6.20	4.86

## Data Availability

Data is contained within the article and Supplementary Materials.

## Supplementary Materials

The following supporting information can be downloaded at: https://www.mdpi.com/article/10.3390/foods11131821/s1, Text S1 [30,31]: SEM and TEM analysis; Figure S1: SEM images of PPP (**A**) and PPP-Cr(III) (**B**) at magnification 5000×; TEM images of PPP (**C**) and PPP-Cr(III) (**D**).

## Abbreviations

AMPK: adenosine 5’-monophosphate (AMP)-activated protein kinase; GSK-3β, glycogen synthase kinase-3β; IRS-1, insulin receptor substrate 1; PI3K, phosphatidylinositide 3-kinases; PKB/Akt, protein kinase B.

## References

[1] Lu A., Yu M., Fang Z., Xiao B., Guo L., Wang W., Li J., Wang S., Zhang Y. (2019). Preparation of the controlled acid hydrolysates from pumpkin polysaccharides and their antioxidant and antidiabetic evaluation. Int. J. Biol. Macromol..

[2] Li F., Wei Y., Liang L., Huang L., Yu G., Li Q. (2021). A novel low-molecular-mass pumpkin polysaccharide: Structural characterization, antioxidant activity, and hypoglycemic potential. Carbohydr. Polym..

[3] Zhao X.H., Qian L., Yin D.L., Zhou Y. (2014). Hypolipidemic effect of the polysaccharides extracted from pumpkin by cellulase-assisted method on mice. Int. J. Biol. Macromol..

[4] Chen L., Huang G. (2019). Antioxidant activities of sulfated pumpkin polysaccharides. Int. J. Biol. Macromol..

[5] Dhenge R., Rinaldi M., Ganino T., Santi S., Ferrarese I., Dall’Acqua S. (2022). Variations of polyphenols, sugars, carotenoids, and volatile constituents in pumpkin (*Cucurbita moschata*) during high pressure processing: A kinetic study. Innov. Food Sci. Emerg. Technol..

[6] Zhou T., Kong Q., Huang J., Dai R., Li Q. (2007). Characterization of nutritional compounds and utilization of pumpkin. Food.

[7] Lima P.M., Dacanal G.C., Pinho L.S., Perez-Cordoba L.J., Thomazini M., Moraes I.C.F., Favaro-Trindade C.S. (2021). Production of a rich-carotenoid colorant from pumpkin peels using oil-in-water emulsion followed by spray drying. Food Res. Int..

[8] Jun H.I., Lee C.H., Song G.S., Kim Y.S. (2006). Characterization of the pectic polysaccharides from pumpkin peel. LWT Food Sci. Technol..

[9] Bai D.S., Ma D., Rui Q., Zhang J. (2012). [Extraction, purification and properties of pumpkin peel polysaccharide PP1]. Food Sci..

[10] Wang Q., Li G.J., Zhou Y.L., Zhao X. (2013). [Chemical composition and antioxidant activity of crude polysaccharides extracted from pumpkin peel as affected by different extraction methods]. Food Sci..

[11] Dey M., Das M., Chowhan A., Giri T.K. (2019). Breaking the barricade of oral chemotherapy through polysaccharide nanocarrier. Int. J. Biol. Macromol..

[12] Gao P., Bian J., Xu S., Liu C., Sun Y., Zhang G., Li D., Liu X. (2020). Structural features, selenization modification, antioxidant and anti-tumor effects of polysaccharides from alfalfa roots. Int. J. Biol. Macromol..

[13] Jia Y., Li N., Wang Q., Zhou J., Liu J., Zhang M., He C., Chen H. (2021). Effect of Fe (III), Zn (II), and Cr (III) complexation on the physicochemical properties and bioactivities of corn silk polysaccharide. Int. J. Biol. Macromol..

[14] Ognik K., Dworzanski W., Sembratowicz I., Fotschki B., Cholewinska E., Listos P., Juskiewicz J. (2021). The effect of the high-fat diet supplemented with various forms of chromium on rats body composition, liver metabolism and organ histology Cr in liver metabolism and histology of selected organs. J. Trace Elem. Med. Biol..

[15] Zhang Y., Luo J., Zhu T., Zhang X., Jin M., Jiao L., Meng F., Figueiredo-Silva C., Hong Y., Zhou Q. (2022). Dietary chromium could improve growth, antioxidant capacity, chromium accumulation in tissues and expression of genes involved into glucose and lipid metabolism in juvenile mud crab *Scylla paramamosain*. Aquacult. Rep..

[16] Wang X., Ye H., Cui J., Chi Y., Liu R., Wang P. (2022). Hypolipidemic effect of chromium-modified enzymatic product of sulfated rhamnose polysaccharide from *Enteromorpha prolifera* in type 2 diabetic mice. Marine Life Sci. Technol..

[17] Guo W.L., Chen M., Pan W.L., Zhang Q., Xu J.X., Lin Y.C., Li L., Liu B., Bai W.D., Zhang Y.Y. (2020). Hypoglycemic and hypolipidemic mechanism of organic chromium derived from chelation of *Grifola frondosa* polysaccharide-chromium (III) and its modulation of intestinal microflora in high fat-diet and STZ-induced diabetic mice. Int. J. Biol. Macromol..

[18] Zhang C., Huang M., Hong R., Chen H. (2019). Preparation of a *Momordica charantia* L. polysaccharide chromium (III) complex and its anti-hyperglycemic activity in mice with streptozotocin-induced diabetes. Int. J. Biol. Macromol..

[19] Li L.Y., Qiu Z.C., Dong H.J., Ma C.X., Qiao Y.T., Zheng Z.J. (2021). Structural characterization and antioxidant activities of one neutral polysaccharide and three acid polysaccharides from the roots of *Arctium lappa* L.: A comparison. Int. J. Biol. Macromol..

[20] Li L., Xu J.X., Cao Y.J., Lin Y.C., Guo W.L., Liu J.Y., Bai W.D., Zhang Y.Y., Ni L., Liu B. (2019). Preparation of *Ganoderma lucidum* polysaccharidechromium (III) complex and its hypoglycemic and hypolipidemic activities in high-fat and high-fructose diet-induced pre-diabetic mice. Int. J. Biol. Macromol..

[21] Saha S.K., Brewer C.F. (1994). Determination of the concentrations of oligosaccharides, complex type carbohydrates, and glycoproteins using the phenol-sulfuric acid method. Carbohydr. Res..

[22] Bradord M.M. (1976). A rapid and sensitive method for the quantitation of microgram quantities of protein utilizing the principle of protein-dye binding. Anal. Biochem..

[23] Guo W.L., Shi F.F., Li L., Xu J.X., Chen M., Wu L., Hong J.L., Qian M., Bai W.D., Liu B. (2019). Preparation of a novel *Grifola frondosa* polysaccharide-chromium (III) complex and its hypoglycemic and hypolipidemic activities in high fat diet and streptozotocin-induced diabetic mice. Int. J. Biol. Macromol..

[24] Yang Y.M., Qiu Z.C., Li L.Y., Vidyarthi S.K., Zheng Z.J., Zhang R.T. (2021). Structural characterization and antioxidant activities of one neutral polysaccharide and three acid polysaccharides from *Ziziphus jujuba* cv. Hamidazao: A comparison. Carbohydr. Polym..

[25] Lu Y.L., Liang J., Zhou F.Y., Kuang H.X., Xia Y.G. (2021). Discrimination and characterization of *Panax* polysaccharides by 2D COS-IR spectroscopy with chemometrics. Int. J. Biol. Macromol..

[26] Chen X.Q., Wu X.F., Zhang K., Sun F.J., Zhou W.L., Wu Z.Q., Li X.T. (2022). Purification, characterization, and emulsification stability of high- and low-molecular-weight fractions of polysaccharide conjugates extracted from green tea. Food Hydrocolloids..

[27] Wang S., Li Y., Huang D.J., Chen S.W., Xia Y.M., Zhu S. (2022). The inhibitory mechanism of chlorogenic acid and its acylated derivatives on alpha-amylase and alpha-glucosidase. Food Chem..

[28] Wu J.S., Huang R., Jiao D.X., Liu S.Y., Liu H.M., Liu H.Z. (2022). Protection by *Hosta ventricosa* polysaccharides against oxidative damage induced by t-BHP in HepG2 cells via the JNK/Nrf2 pathway. Int. J. Biol. Macromol..

[29] Wang Z.C., Sun L.L., Fang Z.X., Nisar T., Zou L., Li D., Guo Y.R. (2021). *Lycium ruthenicum* Murray anthocyanins effectively inhibit α-glucosidase activity and alleviate insulin resistance. Food Biosci..

[30] Dong F., Zheng H.Z., Jeong W.S., Chung S.K., Qu Z.Y., Zou X., Liu C., Xiang Q., Feng F. (2021). Synthesis, characterization, and antioxidant activity in vitro of selenium-*Euryale ferox* Salisb. polysaccharide. Appl. Biol. Chem..

[31] Wang J., Chen H., Wang Y., Xing L. (2015). Synthesis and characterization of a new *Inonotus obliquus* polysaccharide-iron (III) complex. Int. J. Biol. Macromol..

[32] Wang J.L., Zhao B.T., Wang X.F., Yao J., Zhang J. (2012). Synthesis of selenium-containing polysaccharides and evaluation of antioxidant activity in vitro. Int. J. Biol. Macromol..

[33] Li F., Zhao J., Wei Y.L., Jiao X., Li Q.H. (2021). Holistic review of polysaccharides isolated from pumpkin: Preparation methods, structures and bioactivities. Int. J. Biol. Macromol..

[34] Zhang M., Zhao H., Shen Y., Wang Y.L., Zhao Z.M., Zhang Y. (2020). Preparation, characterization and antioxidant activity evaluation in vitro of *Fritillaria ussuriensis* polysaccharide-zinc complex. Int. J. Biol. Macromol..

[35] Rao C.P., Kaiwar S.P. (1992). Chromate reduction: Reduction of potassium chromate by D-glucose and D-fructose to form Cr(III)-saccharide complexes. Carbohydr. Res..

[36] Geetha K., Raghavan M.S.S., Kulshreshtha S.K., Sasikala R., Rao C.P. (1995). Transition-metal saccharide chemistry synthesis, spectroscopy, electrochemistry and magnetic susceptibility studies of iron(Ill)complexes of mono- and disaccharides. Carbohydr. Res..

[37] Yuan L.L., Qiu Z.C., Yang Y.M., Liu C., Zhang R.T. (2022). Preparation, structural characterization and antioxidant activity of water-soluble polysaccharides and purified fractions from blackened jujube by an activity-oriented approach. Food Chem..

[38] Ranjbar B., Gill P. (2009). Circular dichroism techniques: Biomolecular and nanostructural analyses—A review. Chem. Biol. Drug. Des..

[39] Park J.W., Chakrabarti B. (1978). Optical characteristic of carboxyl group in relation to the circular dichroic properties and dissociation constants of glycosaminoglycans. Biochim. Biophys. Acta..

[40] Zhao M.M., Bai J.W., Bu X.Y., Yin Y.T., Wang L.B., Yang Y., Xu Y.Q. (2021). Characterization of selenized polysaccharides from *Ribes nigrum* L. and its inhibitory effects on alpha-amylase and alpha-glucosidase. Carbohydr. Polym..

[41] Wang C., Chen Z.Q., Pan Y.X., Gao X.D., Chen H.X. (2017). Anti-diabetic effects of *Inonotus obliquus* polysaccharides-chromium (III) complex in type 2 diabetic mice and its sub-acute toxicity evaluation in normal mice. Food Chem. Toxicol..

[42] Xiong G.Y., Ma L.S., Zhang H., Li Y.P., Zou W.S., Wang X.F., Xu Q.S., Xiong J.T., Hu Y.P., Wang X.Y. (2022). Physicochemical properties, antioxidant activities and alpha-glucosidase inhibitory effects of polysaccharides from *Evodiae fructus* extracted by different solvents. Int. J. Biol. Macromol..

[43] Fu C.Y., Ren L., Liu W.J., Sui Y., Nong Q.N., Xiao Q.H., Li X.Q., Cao W. (2021). Structural characteristics of a hypoglycemic polysaccharide from *Fructus Corni*. Carbohydr. Res..

[44] Song J.J., Gao J., Du M., Mao X.Y. (2018). Casein glycomacropeptide hydrolysates ameliorate hepatic insulin resistance of C57BL/6J mice challenged with high-fat diet. J. Funct. Foods..

[45] Ye H., Shen Z.P., Cui J.F., Zhu Y.J., Li Y.Y., Chi Y.Z., Wang J.F., Wang P. (2019). Hypoglycemic activity and mechanism of the sulfated rhamnose polysaccharides chromium(III) complex in type 2 diabetic mice. Bioorg. Chem..

[46] Chen L., Lin X.J., Fan X.Y., Qian Y.W., Lv Q.Y., Teng H. (2020). *Sonchus oleraceus* Linn extract enhanced glucose homeostasis through the AMPK/Akt/GSK-3β signaling pathway in diabetic liver and HepG2 cell culture. Food Chem. Toxicol..

[47] Wu Y., Li W., Cui W., Eskin N.A.M., Goff H.D. (2012). A molecular modeling approach to understand conformation–functionality relationships of galactomannans with different mannose/galactose ratios. Food Hydrocolloids.

[48] Chen S., Khan B.M., Cheong K.L., Liu Y. (2019). Pumpkin polysaccharides: Purification, characterization and hypoglycemic potential. Int. J. Biol. Macromol..

[49] Deng Y.J., Huang L.X., Zhang C.H., Xie P.J., Cheng J., Wang X., Liu L.J. (2020). Novel polysaccharide from *Chaenomeles speciosa* seeds: Structural characterization, α-amylase and α-glucosidase inhibitory activity evaluation. Food Biosci..

[50] Xu Y.Q., Guo Y.Y., Duan S.Y., Wei H., Liu Y.S., Wang L.B., Huo X., Yang Y. (2018). Effects of ultrasound irradiation on the characterization and bioactivities of the polysaccharide from blackcurrant fruits. Ultrason. Sonochem..

[51] Hoffman N.J., Penque B.A., Habegger K.M., Sealls W., Tackett L., Elmendorf J.S. (2014). Chromium enhances insulin responsiveness via AMPK. J. Nutr. Biochem..

[52] Deng J., Zhong J., Long J., Zou X.Y., Wang D., Song Y., Zhou K., Liang Y.X., Huang R.M., Wei X.Q. (2020). Hypoglycemic effects and mechanism of different molecular weights of konjac glucomannans in type 2 diabetic rats. Int. J. Biol. Macromol..

