# Supplementary material for: Structural Characterization and Hypoglycemic Activity of a Novel Pumpkin Peel Polysaccharide-Chromium(III) Complex
Source: Foods. 2022 Jun 21;11(13):1821. doi: 10.3390/foods11131821 (PMC9265534; doi:10.3390/foods11131821)
Supplement: Supplementary file 1 [file foods-11-01821-s001.zip › foods-1745539-supplementary.pdf]

Supplementary Data

Structural characterization and hypoglycemic activity of a novel pumpkin peel polysaccharide-chromium(III) complex

Wen Zhang [1,†], Lingyu Li [1,†], Yue Ma [2], Xiaole Chen [1], Tao Lan [3,*], Long Chen [4], and Zhenjia Zheng [1,*]

1 Key Laboratory of Food Processing Technology and Quality Control of Shandong Higher Education Institutes, College of Food Science and Engineering, Shandong Agricultural University, 61 Daizong Street, Tai'an 271018, P.R. China; 18364705726@163.com (W.Z.); sdnydxlly@163.com (L.L.); c18854881522@163.com (X.C.)

2 Institute of Agri-food Processing and Nutrition, Beijing Academy of Agriculture and Forestry Sciences, Beijing Key Laboratory of Agricultural Products of Fruits and Vegetables Preservation and Processing, Key Laboratory of Vegetable Postharvest Processing, Ministry of Agriculture and Rural Affairs, Beijing 100097, PR China; mayue@iapn.org.cn

3 Sub-Institute of Agriculture and Food Standardization, China National Institute of Standardization, Beijing 100191, PR China

4 Key Laboratory for Applied Technology of Sophisticated Analytical Instruments of Shandong Province, Shandong Analysis and Test Center, Qilu University of Technology (Shandong Academy of Sciences), Jinan 250014, PR China; chlong718@126.com

* Correspondence: lantao@cnis.ac.cn (T.L.); pengyou-jia@163.com (Z.Z.); Tel.: +86-0105-8811108 (T.L.); +86-0538-8242875 (Z.Z.)

† These authors contributed equally to this work

Text S1. SEM and TEM analysis

SEM and TEM are powerful tools to evaluate the difference in the surface and internal morphological structure between PPP and PPP-Cr(III). The surface morphological characterization was analyzed by a ZEISS-SUPRA™ 55 scanning electron microscopy (Carl Zeiss, Germany). Powdered samples were fixed on the sample stage and then coated with a thin layer of gold. The shapes and surface characteristics of PPP and PPP-Cr(III) were observed at an acceleration voltage of 5.0 kV under a high vacuum condition. The TEM images were recorded by an FEI Talos™ F200s instrument (Thermo Fisher Scientific Inc., USA). Briefly, 1 mg of the samples were diluted by 10 mL of ethanol solution, followed by ultrasonication at 100 W for 30 min. Then, 10 μL of sample suspension was dropped onto a copper mesh and treated in a desiccator at 20 °C for 2 h. The obtained dried samples in the copper mesh were used for TEM analysis.

SEM images showed an obvious difference in surface morphology between PPP and PPP-Cr(III). As shown in Figure S1A and Figure S1B PPP was granules with a rough surface, while PPP-Cr(III) had a thin sheet-like structure with smooth surfaces. Besides, the structure of PPP-Cr(III) was dense, indicating that there existed strong inter molecular attraction [30]. These morphology changes between PPP and PPP-Cr(III) may be that there was increased inter molecular PPP-Cr(III) cross-linking in the coordinated process of PPP and Cr(III) [31]. Besides, the fine structures were confined by TEM images. Wherein, PPP was the sheet-like structure (Figure S1C), and PPP-Cr(III) was the sheet-like structure with nanoparticles (Figure S1D). Therefore, we inferred that the nanoparticles were Cr(III) compounds. The TEM images revealed that all nanoparticles were decorated on the PPP nanosheets in the PPP-Cr(III) composite, and no individual Cr(III) were observed (Figure S1D), which showed that all of Cr(III) has been loaded onto the PPP.

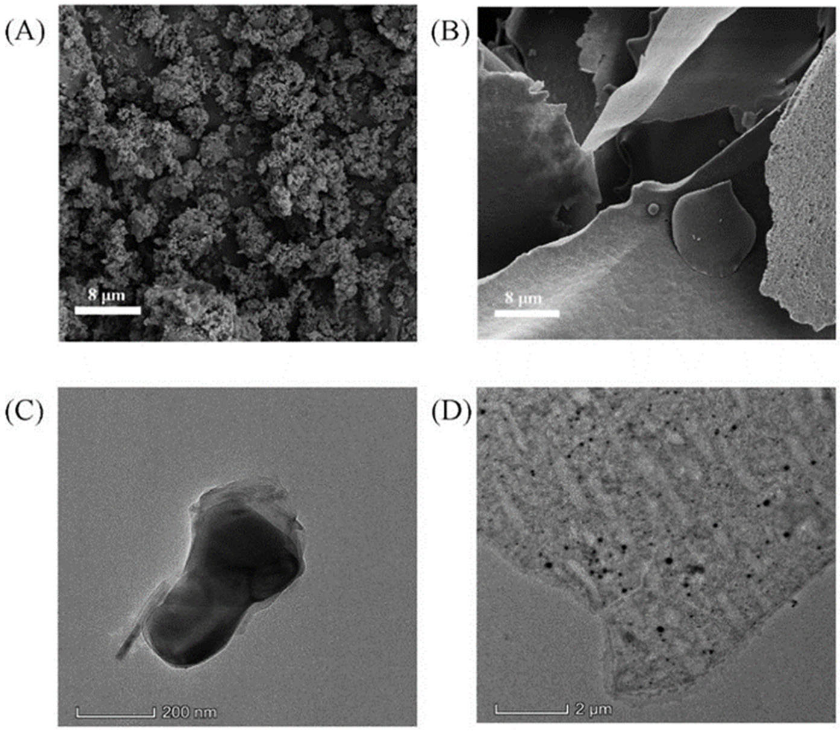

Figure S1. SEM images of PPP (A) and PPP-Cr(III) (B) at magnification 5000×; TEM images of PPP (C) and PPP-Cr(III) (D).

Reference

30 Dong, F.; Zheng, H.Z.; Jeong, W.S.; Chung, S.K.; Qu, Z.Y.; Zou, X.; Liu C.; Xiang, Q.; Feng, F. Synthesis, characterization, and antioxidant activity *in vitro* of selenium-*Euryale ferox Salisb.* polysaccharide. *Appl. Biol. Chem*. 2021, *64*, 59. http://doi.org/10.1186/s13765-021-00631-4.

31 Wang, J.; Chen, H.; Wang, Y.; Xing, L. Synthesis and characterization of a new *Inonotus obliquus* polysaccharide-iron (III) complex. *Int. J. Biol. Macromol.* **2015**, *75*, 210–217. http://doi.org/10.1016/j.ijbiomac.2015.01.041.
